# Sulphonamide inhibition profile of *Staphylococcus aureus* β-carbonic anhydrase

**DOI:** 10.1080/14756366.2020.1826942

**Published:** 2020-09-24

**Authors:** Linda J. Urbanski, Silvia Bua, Andrea Angeli, Marianne Kuuslahti, Vesa. P. Hytönen, Claudiu T. Supuran, Seppo Parkkila

**Affiliations:** a Faculty of Medicine and Health Technology, Tampere University, Tampere, Finland; b Neurofarba Department, Sezione di Chimica Farmaceutica e Nutraceutica, Università degli Studi di Firenze, Sesto Fiorentino (Firenze), Italy; c Fimlab Ltd, Tampere University Hospital, Tampere, Finland

**Keywords:** β-carbonic anhydrase, *Staphylococcus aureus*, kinetics, inhibition, sulphonamides

## Abstract

This paper presents the production and kinetic and inhibitory characterisation of β-carbonic anhydrase from the opportunistic bacterium *Staphylococcus aureus* (SauBCA). From the eight different carbonic anhydrase (CA) families known to date, humans have only the α-form, whereas many clinically relevant pathogens have β- and/or γ-form(s). Based on this discovery, β- and γ-CAs have been introduced as promising new anti-infective targets. The results of this study revealed that recombinant SauBCA possesses significant CO_2_ hydration activity with a *k_cat_* of 1.46 × 10^5^ s^−1^ and a *k_cat_/K_M_* of 2.56 × 10^7^ s^− 1^M^−1^. Its enzymatic function was inhibited by various sulphonamides in the nanomolar − micromolar range, and the *K_i_* of acetazolamide was 628 nM. The best inhibitor was the clinically used sulfamide agent famotidine (*K_i_* of 71 nM). The least efficient inhibitors were zonisamide and dorzolamide. Our work encourages further investigations of SauBCA in an attempt to discover novel drugs against *staphylococcal* infections.

## Introduction

*Staphylococcal* species represent a significant part of our microbiota, e.g. on our skin and in our mouth and gut^1–3^. Most of these species are harmless, commensal bacteria that do not cause inflammation (e.g. *S. epidermidis*)^3^. However, one particular strain of *staphylococci* has been at the centre of attention since our first encounter with it over a century ago^4^. Despite huge improvements in health care, *Staphylococcus aureus* has caused increasing morbidity and, in some cases, mortality^4^^,^^5^. Due to the emergence of multi-drug-resistant strains (collectively termed MRSA, i.e. methicillin-resistant *S. aureus*), treatment has remained particularly challenging^4^. The first nosocomial MRSA emerged not too long after Ian Fleming discovered penicillin^6^^,^^7^, and more strains have emerged, causing it to be one of the most prominent causative agents of surgical-site infections and invasive bacterial diseases^8–10^. In the past 20 years, *S. aureus* infections have increased dramatically, causing an increase in MRSA strains displaying resistance to penicillin-derived β-lactam antibiotics^11^^,^^12^. The antibiotic resistance of MRSA is based on the single gene mecA, which encodes a penicillin inactivating enzyme on the surface of the bacteria^13^^,^^14^. In addition, the virulence of *staphylococci* is based on their ability to attach to foreign bodies by specialised adhesins and form a biofilm to shield itself from antibiotics^15^. MRSA is particularly resilient in hospital settings, where patients with diseases such as type I diabetes, immunodeficiencies or ongoing haemodialysis are at the greatest risk of acquiring the infection^12^^,^^16^^,^^17^. Most cases occur through hospital personnel who are infected by their own reservoir or by infected patients^1^^,^^18^. The infection requires physical contact and is activated when the skin or mucosal barrier is breached, allowing the bacteria to enter adjoining tissues or the bloodstream^4^. Infections vary from mild skin lesions to severe cases of sepsis, endocarditis, osteomyelitis and pneumonia^4^. Not all individuals show signs of *S. aureus* infection; hence, it can easily be passed on unnoticed via the skin in addition to from surgical instrumentation and other inanimate surfaces^15^^,^^19^.

Novel innovative therapies are not only needed but also quickly becoming a necessity as the number of antibiotic-resistant pathogen strains increases. Scientists worldwide are thus exploring novel strategies for the prevention of these most threatening pathogenic infections. Among the promising biomolecular targets are carbonic anhydrases (CAs), a group of metalloenzymes found in all lifeforms. These vitally important enzymes catalyse a reversible reaction in which CO_2_ is converted to bicarbonate ions and protons. This simple reaction is responsible for numerous vital cellular functions, such acid-base homeostasis, CO_2_ transportation, and photosynthesis^20–22^. Among the eight evolutionarily divergent but functionally convergent *CA* gene families (α, β, γ, δ, ζ, η, θ, and ι), humans have only the α-forms^23^^,^^24^. Interestingly, numerous pathogens have been identified with only *β-* and/or *γ-CA* genes in their genome. This has been regarded as a promising starting point for discovering novel, specific anti-infectives against pathogens. The fundamental differences discovered between the active sites of different CA families support the idea of specifically targeting β- and/or γ-CAs with minimal effects on human α-CAs. Numerous studies have been conducted demonstrating the efficiency of sulphonamides and anions as CA inhibitors (CAIs)^25–31^. Detailed characterisation of druggable CAs is considered a prerequisite for the design of more specific and efficient CAIs. To date, several crystal structures of pathogenic β-CAs have been solved and can consequently be exploited for more efficient CAI design^32–39^. Such structure-based design allows for *in vivo* targeted therapeutics against pathogenic diseases without the disadvantage of affecting the human or other mammalian CAs.

In the present study, we produced and isolated a novel β-CA from *S. aureus* (SauBCA) as a recombinant protein and tested its kinetic properties and inhibition profile against several known sulphonamides. Our results demonstrate that SauBCA represents a druggable enzyme target that should be further tested both *in vitro* and *in vivo* using different classes of potential CA inhibitors.

## Materials and methods

### Protein production

The *SauBCA* gene, obtained from the Universal Protein Resource Database (UniProt, protein entry EZX15767^40^), was cloned into the expression vector pBVboostFG^41^ by GeneArt (Thermo Fisher Scientific, Germany). The synthesised insert was composed of Gateway-compatible recombination sites (attL1 and attL2), Shine-Dalgarno and Kozak sequences, N-terminal 6× His-tag with surrounding spacer regions (MSTT and ATAIPTT^42^), *SauBCA*, and a thrombin cleavage site (LVPRGS)^43^. Chemically competent *E. coli* (OneShot® BL21 Star™ (DE3) cells, #C601003, Thermo Fisher Scientific) were transformed according to the Thermo Fisher Scientific OneShot® BL21(DE3) competent cells manual (part no. 28–0182). The culture medium used was Luria-Bertani (LB) supplemented with 10 mg/mL gentamicin (1:1000, v/v). The cells were grown in a fermenter at 28 °C for 12 h and subsequently induced by adding 1 mM isopropyl β-D-1-isopropyl-thiogalactopyranoside (IPTG). After another 12 h of culture at 25 °C, the cells were harvested by centrifugation at 4000 g for 40 min at 4 °C and mechanically disrupted with an EmulsiFlex-C3 homogeniser (AVESTIN, Canada). Subsequently, the cells were centrifuged at 13000 g for 20 min at 4 °C, and the supernatant was mixed with Protino® Nickel-nitrilotriacetic acid (Ni^2+^-NTA) agarose affinity chromatography resin (Macherey-Nagel GmbH Co., Germany) and 50 mM Na_2_HPO_4_, 0.5 M NaCl and 50 mM imidazole binding buffer (BB; pH 8.0). The incubation took place for 2 h at room temperature (RT) with gentle agitation, followed by overnight incubation at 4 °C without agitation. The resin was loaded into a chromatography column with an EMD Millipore™ vacuum filtering flask (Merck, #XX1004705) and washed generously with BB. The protein was eluted from the column with 50 mM Na_2_HPO_4_, 0.5 M NaCl and 350 mM imidazole (pH 7.0). After elution, the eluted fractions were analysed with reducing sodium dodecyl sulphate gel electrophoresis (SDS-PAGE) using a 12% (w/v) polyacrylamide gel and visualised with PageBlue protein staining solution (Thermo Fisher Scientific, #24620). The polypeptide bands on SDS-PAGE were used to identify the protein by means of tandem mass spectrometry (MS/MS, Meilahti Clinical Proteomics Core Facility, University of Helsinki, Finland). A 6× His-tag was enzymatically cleaved using thrombin (#RECOMT, Sigma-Aldrich) according to the Thrombin CleanClive™ kit manual instructions (Sigma-Aldrich). The tag was separated from the core protein with Ni^2+-^NTA affinity chromatography as mentioned above. Prior to further characterisation, the buffer was exchanged for 50 mM Tris-Cl (pH 7.5).

### Kinetics and inhibition studies

An Applied Photophysics stopped-flow instrument was used for assaying CA-catalysed CO_2_ hydration activity^44^. Phenol red (at a concentration of 0.2 mM) was used as the pH indicator at the absorbance maximum of 557 nm with 20 mM TRIS (pH 8.4) as the buffer with 20 mM Na_2_SO_4_ (for maintaining constant ionic strength) and following the initial rates of the CA-catalysed CO_2_ hydration reaction for over the period of 10 − 100 s. The CO_2_ concentrations ranged from 1.7 to 17 mM for the determination of the kinetic parameters and sulphonamide inhibition constants. Six traces of the initial 5 − 10% of the reaction were used to determine the initial velocity. The uncatalysed rates were determined in the same manner and subtracted from the total observed rates. A stock solution of inhibitor (0.1 mM) was prepared in distilled-deionized water, and dilutions of up to 0.01 nM were prepared thereafter with distilled-deionized water. Inhibitor (I) and enzyme (E) solutions were preincubated together for 15 min at RT prior to the assay to allow formation of the E − I complex. Inhibition constants were obtained by using the Cheng-Prusoff equation and nonlinear least squares methods (with PRISM 3) and are presented as the means from at least three different determinations. Kinetic constants were obtained using Lineweaver-Burke plots as reported earlier^45–47^.

## Results and discussion

### Protein production

**Figure 1.**
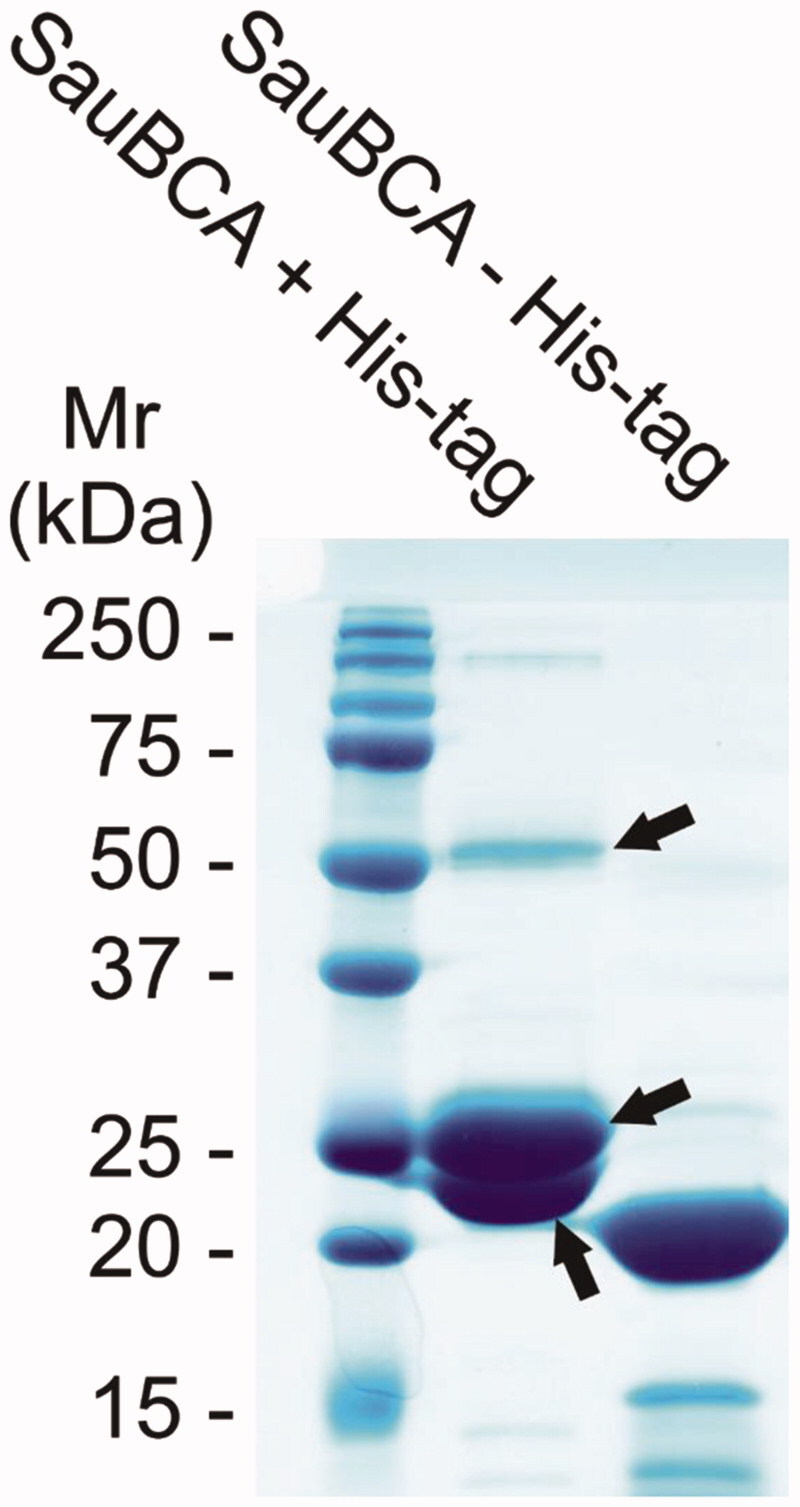
SDS-PAGE of the purified SauBCA with and without the His-tag. The polypeptides marked with arrows were subjected to MS/MS analysis and identified as SauBCA.

A single β-CA enzyme of *S. aureus* was identified from the UniProt database^40^ and named SauBCA. The enzyme was successfully expressed in *E. coli* and purified by affinity chromatography. SDS-PAGE was used to monitor the purification process and successful cleavage of the His-tag. The tag was cleaved prior to further characterisation experiments. The gel from SDS-PAGE was stained with PageBlue and is shown in Figure 1. The relative molecular mass of the major band seen on the gel after His-tag removal corresponded to approximately 21 kDa. The theoretical molecular mass of SauBCA was calculated to be 21.1 kDa, suggesting that the purification procedure yielded the correct protein. This was further confirmed by MS/MS analysis, where three major polypeptides were analysed and identified as SauBCA. The relatively strong ∼52 kDa polypeptide seemed to represent a dimeric form of the protein.

### Kinetics

The obtained kinetic parameters for SauBCA are shown in Table 1 together with human α-CA isoforms and other representative β-CAs for comparison. Kinetic analysis of SauBCA revealed that it is a moderately efficient enzyme with a *k_cat_* of 1.46 × 10^5^ s^−1^ and *k_cat_/K_M_* of 2.56 × 10^7^ s^−1^M^−1^. SauBCA showed kinetic properties fairly similar to several human CA isozymes, such as CA I, CA VA, CA VI, CA XII, CA XIII, and CA XIV. Notably, the *k_cat_* value of SauBCA was identical to that of hCA XIII, whereas it was the most different from the clinically relevant isozymes hCA II and hCA IX, as well as to the low activity enzyme hCA III. Similar kinetics were observed with the β-CAs from *Burkholderia pseudomallei* and *Flaveria bidentis*. As seen in Table 1, the binding affinities of SauBCA and *Burkholderia pseudomallei* β-CA to acetazolamide (AAZ) are rather similar, whereas the inhibition constant for *Flaveria bidentis* β-CA is very different. This finding suggests major structural differences in the active sites of these β-CA enzymes.

**Table 1.** Kinetic data of SauBCA and the inhibition results for the standard sulphonamide inhibitor acetazolamide (AAZ). All human isozymes^22^^,^^48^ and two other β-CAs^26^^,^^27^^,^^49^ with corresponding properties are shown for comparison. The *k_cat_* and *k_cat_/K_M_* values are rounded to one decimal place.

Enzyme	*k_cat_*	*k_cat_/K_M_*	*K_i_* (AAZ)
	(s-1)	(s-1M-1)	(nM)
SauBCA	1.5 × 10^5^	2.6 × 10^7^	628
hCA I	2.0 × 10^5^	5.0 × 10^7^	250
hCA II	1.4 × 10^6^	1.5 × 10^8^	12
hCA III	1.3 × 10^4^	2.5 × 10^5^	240000
hCA IV	1.1 × 10^6^	5.1 × 10^7^	74
hCA VA	2.9 × 10^5^	2.9 × 10^7^	63
hCA VB	9.5 × 10^5^	9.8 × 10^7^	54
hCA VI	3.4 × 10^5^	4.9 × 10^7^	11
hCA VII	9.5 × 10^5^	8.3 × 10^7^	2.5
hCA IX	1.1 × 10^6^	1.5 × 10^8^	16
hCA XII	4.2 × 10^5^	3.5 × 10^7^	5.7
hCA XIII	1.5 × 10^5^	1.1 × 10^7^	16
hCA XIV	3.1 × 10^5^	3.9 × 10^7^	41
*Burkholderia pseudomallei* β-CA	1.6 × 10^5^	3.4 × 10^7^	745
*Flaveria bidentis* β-CA	1.2 × 10^5^	7.5 × 10^6^	27

### Inhibition studies

**Figure 2.**
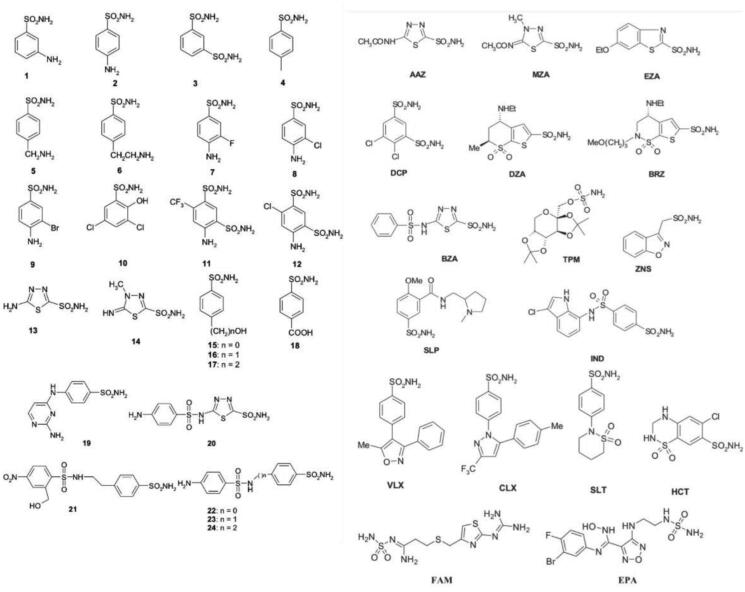
The molecular structures of the sulphonamide analogues used in this study (**1–24**) as well as selected clinically used agents.

A set of clinically used sulphonamide drugs and sulphonamide analogues were investigated against SauBCA. The obtained inhibition constants can be seen in Table 2, along with the results from human α-CA isoform II for comparison. The molecular structures of sulphonamides **1–24** and of the clinically used agents tested in this study are shown in Figure 2.

**Table 2.** Inhibition data for SauBCA and hCA II with sulphonamide analogues **1–24** and selected clinically used agents.

K_i_* (nM)		
Inhibitor	SauBCA	hCA II^a^
**1**	355	300
**2**	409	240
**3**	95	8
**4**	83	320
**5**	193	170
**6**	253	160
**7**	93	60
**8**	95	110
**9**	75	40
**10**	202	54
**11**	81	63
**12**	79	75
**13**	417	60
**14**	553	19
**15**	619	80
**16**	603	94
**17**	232	125
**18**	555	46
**19**	909	33
**20**	92	2
**21**	85	11
**22**	83	46
**23**	92	33
**24**	96	30
**AAZ**	628	12
**MZA**	863	14
**EZA**	698	8
**DZA**	909	9
**BRZ**	815	3
**BZA**	501	9
**TPM**	466	10
**ZNS**	4551	35
**SLP**	807	40
**IND**	588	15
**VLX**	509	43
**CLX**	871	21
**SLT**	824	9
**SAC**	667	5959
**HCT**	593	290
**FAM**	71	58
**EPA**	538	917

*Mean from three different assays measured by the stopped-flow technique. Errors were in the range of ±5–10% of the reported values (data not shown). ^a^Human recombinant isozyme, from Ref.^22^.

SauBCA was successfully inhibited by selected sulphonamide analogues **1–24** and standard sulphonamide inhibitors **AAZ-**epacadostat **(EPA)** in the nanomolar range. In general, all studied compounds resulted in strong to medium *K_i_* values spanning between 71 and 4551 nM. The most efficient inhibition was obtained with sulphonamide analogues **9, 11, 12, 21**, **22, 24** and famotidine (**FAM**). Their inhibition affinities were < 100 nM, which indicates strong inhibition against the SauBCA catalytic function. The least efficient inhibitors were the following sulphonamides: zonisamide (ZNS), dorzolamide (**DZA**), **19**, celecoxib (**CLX**), methazolamide (**MZA**), sulthiame (**SLT**), and brinzolamide (**BRZ**). Despite showing the lowest efficiency in this study, their inhibition can still be regarded as medium/medium-weak. **AAZ** ranked 29th out of 41 studied sulphonamides with a *K_i_* of 628 nM. In general, the clinically used sulphonamides showed inferior inhibition compared to designed sulphonamide analogues **1–24**, except for **FAM,** which was in fact the most effective inhibitor. However, **FAM** (together with **EPA**) are the only sulfamide derivatives in the series, and FAM has a quite diverse scaffold compared to the other clinically used drugs investigated here. It has a large aliphatic fragment present in the structure, which is absent in the other compounds investigated in this study. Future work entails the design of more potent inhibitors against SauBCA.

## Conclusion

In the current study, we successfully produced recombinant SauBCA and investigated its kinetics and inhibition profile with sulphonamides. The data showed that SauBCA possesses significant catalytic activity with a *k_cat_* of 1.46 × 10^5^ s^−1^ and *k_cat_/K_M_* of 2.56 × 10^7^ s^−1^ M^−1^. The successful inhibition of SauBCA with selected sulphonamides in the nanomolar range warrants further investigation of potent SauBCA inhibitors for developing future anti-infective agents against *staphylococcal* infections. One of the most effective inhibitors detected in the study was famotidine, a clinically used antiulcer drug, with a *K_i_* of 71 nM.
